# Time-dependent upregulation of electron transport with concomitant induction of regulated excitation dissipation in *Haslea* diatoms

**DOI:** 10.1007/s11120-018-0508-x

**Published:** 2018-04-16

**Authors:** R. Perkins, C. Williamson, J. Lavaud, J.-L. Mouget, D. A. Campbell

**Affiliations:** 1 School of Earth and Ocean Sciences, Cardiff University, Park Place, Cardiff, Wales CF10 3AT UK; 2 Schools of Biological and Geographical Sciences, University of Bristol, 12 Berkeley Square, Bristol, BS8 1SS UK; 3 UMI 3376 Takuvik, CNRS/Université Laval, Département de Biologie-Pavillon Alexandre Vachon, Québec, QC G1V 0A6 Canada; 4 Mer-Molécules-Santé (MMS), FR CNRS 3473 IUML, Le Mans Université, Av. O. Messiaen, 72085 Le Mans Cedex 9, France; 5 Department of Biology, Mount Allison University, Sackville, NB E4L3M7 Canada

**Keywords:** Photoacclimation, Diatom photophysiology, Downregulation, *Haslea*, Electron transport rate

## Abstract

Photoacclimation by strains of *Haslea* “blue” diatom species *H. ostrearia* and *H. silbo* sp. nov. ined. was investigated with rapid light curves and induction–recovery curves using fast repetition rate fluorescence. Cultures were grown to exponential phase under 50 µmol m^−2^ s^−1^ photosynthetic available radiation (PAR) and then exposed to non-sequential rapid light curves where, once electron transport rate (ETR) had reached saturation, light intensity was decreased and then further increased prior to returning to near growth light intensity. The non-sequential rapid light curve revealed that ETR was not proportional to the instantaneously applied light intensity, due to rapid photoacclimation. Changes in the effective absorption cross sections for open PSII reaction centres (σ_PSII_′) or reaction centre connectivity (ρ) did not account for the observed increases in ETR under extended high light. σ_PSII_′ in fact decreased as a function of a time-dependent induction of regulated excitation dissipation Y(NPQ), once cells were at or above a PAR coinciding with saturation of ETR. Instead, the observed increases in ETR under extended high light were explained by an increase in the rate of PSII reopening, i.e. Q_A_^−^ oxidation. This acceleration of electron transport was strictly light dependent and relaxed within seconds after a return to low light or darkness. The time-dependent nature of ETR upregulation and regulated NPQ induction was verified using induction–recovery curves. Our findings show a time-dependent induction of excitation dissipation, in parallel with very rapid photoacclimation of electron transport, which combine to make ETR independent of short-term changes in PAR. This supports a selective advantage for these diatoms when exposed to fluctuating light in their environment.

**Electronic supplementary material:**

The online version of this article (10.1007/s11120-018-0508-x) contains supplementary material, which is available to authorised users.

## Introduction

Variability in the light environment is likely a key parameter dictating diatom species distribution (Lavaud et al. 2007), and hence to our understanding of the ability of diatoms to acclimate to changes in their light environment. Formerly considered to be restricted to a single species, *Haslea ostrearia*, the *Haslea* genus of pennate diatoms has recently been elucidated to comprise > 20 species, occurring globally throughout temperate to tropical ecosystems (Gastineau et al. 2014). This is typical of many taxa of diatoms, with apparent plasticity of environmental preference (Underwood and Kromkamp 1999; Vanormelingen et al. 2008; Malviya et al. 2016). Species of *Haslea* diatoms are commonly referred to as “blue” diatoms due to their production of the blue pigment marennine (e.g. Mouget and Tremblin 2002; Mouget et al. 2004, 2005), which has potential for biotechnological exploitation. Therefore, information on light acclimation mechanisms in microalgae is also relevant in optimising bioreactor light environments (Eriksen 2008; Carvalho et al. 2011; Sforza et al. 2012).

In this study, we investigated photoacclimation in the form of both non-photochemical downregulation used to dissipate excess excitation and inducible upregulation of electron transport rate (ETR) in two species of *Haslea* diatoms, *H. ostrearia* and *H. silbo* sp. nov. ined. (here after *H. silbo*). We follow the definition of MacIntyre et al. (2002), that photoacclimation is a plastic response to a change in light field that adjusts the balance between excitation capture and metabolism (MacIntyre et al. 2002). Excitation dissipation refers to photoprotective processes that act to quench excess excitation pressure upon photosystems and dissipate excess absorbed light energy as heat and/or balance excitation energy within the photosynthetic apparatus (Lavaud and Lepetit 2013). Non-photochemical quenching (NPQ) is one form of excitation dissipation that quenches excitation through conversion of a proportion of antennae complex excitation to heat (Lavaud and Goss 2014). NPQ is often separated into regulated (Y[NPQ]) and non-regulated (Y[NO]) components (Klughammer and Schreiber 2008). Regulated excitation dissipation (Klughammer and Schreiber 2008) includes xanthophyll cycle-mediated processes, through the enzymatic de-epoxidation of diadinoxanthin to diatoxanthin (in diatoms) activated in response to light-induced energisation (proton gradient) across the thylakoid membrane (Ting and Owens 1993; Demmig-Adams and Adams 2006; Lavaud and Goss 2014). Diatoms have a high capacity for excitation dissipation through combinations of both regulated and non-regulated mechanisms (Lavaud et al. 2002, 2007; Lepetit et al. 2012). Regulated excitation dissipation as measured in the form of Stern–Volmer NPQ, has been observed to be kinetically biphasic, with Lavaud and Goss (2014) suggesting the de-epoxidation step as the rapid phase of NPQ, followed by dissociation of fucoxanthin–chlorophyll protein complexes from PSII as the slower phase. Upregulation of the relative electron transport rate (rETR) by diatoms has been reported within rapid light curve (RLC) measurements, where an increase in rETR occurred as a result of rapid photoacclimation to the immediate light history applied during the RLC itself (Perkins et al. 2006, 2010; Lefebvre et al. 2011). The mechanisms underlying this rapid photoacclimation are not fully understood (Perkins et al. 2010), in part because rETR is based solely on changes in the quantum yield of the pool of PSII, without considering possible changes in the effective absorption cross section for photochemistry, or changes downstream of electron transport.

**Fig. 1:**
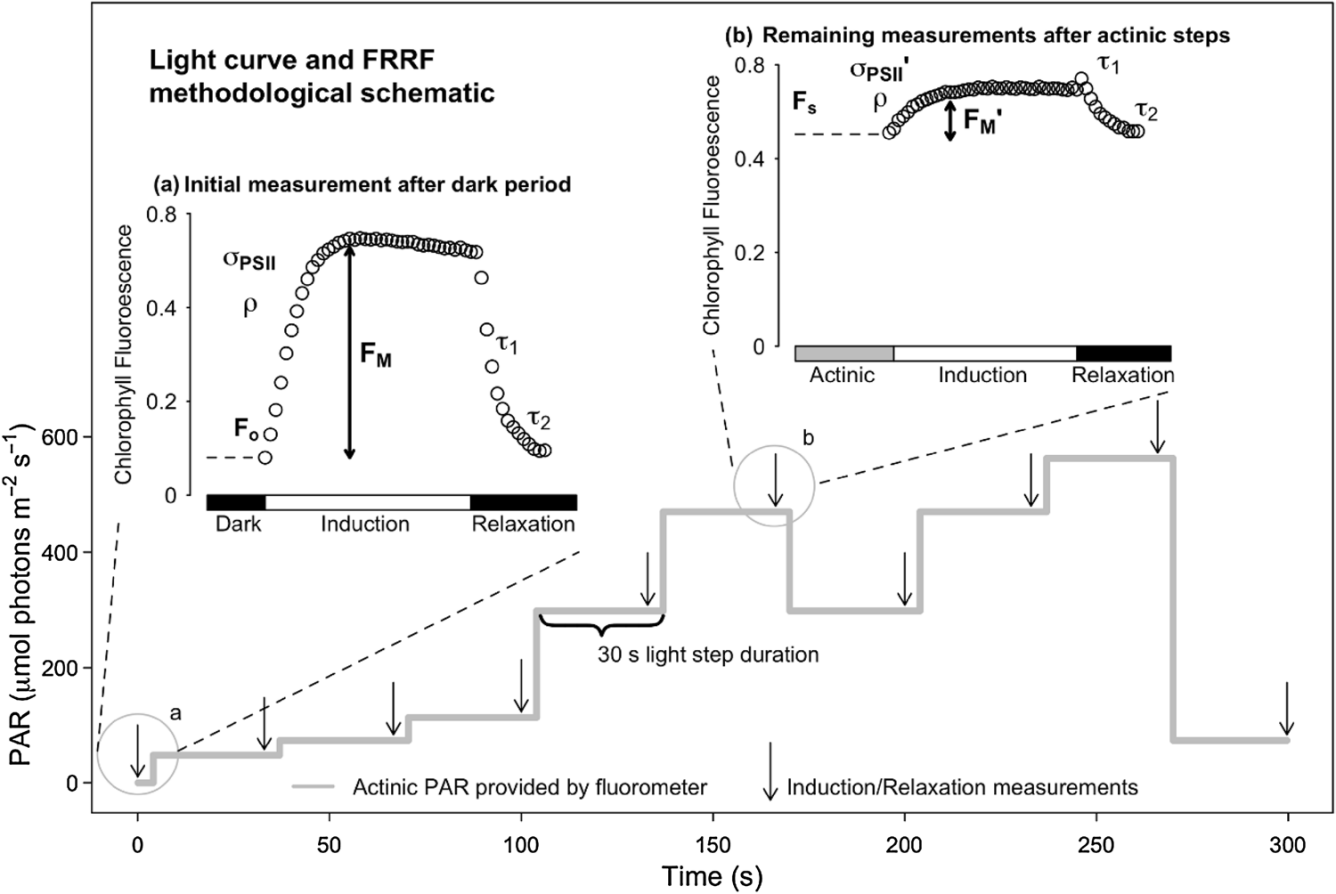
Non-sequential rapid light response treatment and measuring protocol. On the *Y* axis we plot the sequence of light levels applied for 30 s each. Arrowheads show applications of repeated Fast Repetition and Relaxation chlorophyll fluorescence (FRRf) measurements, as demonstrated in annotated inset graphs. The initial FRRf measurement (inset *a*) was performed following dark adaptation of samples, with all subsequent measurements (inset *b*) performed following 30 s at respective light levels. For each measurement, a fast fluorescence induction and relaxation measurement were applied both in the presence of background actinic light (insets), and following 1 s darkness (not shown) immediately after exposure to the preceding light step, to allow reopening of photosystem II

We therefore applied a combined RLC and induction–recovery curve methodology (Ralph and Gademann 2005; Serôdio et al. 2005a; Perkins et al. 2006, 2010) while monitoring excitation capture, photochemistry and electron transfer using fast repetition rate fluorescence (FRRf) (Kolber et al. 1998) to analyse rapid changes in photophysiology and excitation dissipation for the two *Haslea* species. FRRf single turnover methodology generates fluorescence rise (induction) and reaction centre reopening (relaxation) curves that enable estimation of the effective absorption cross section for photochemistry (σ_PSII_ or σ_PSII_′); reaction centre connectivity (ρ or ρ′); the rapid and slow kinetic phase lifetimes for PSII reopening (τ_1_ and τ_2_, respectively), along with the minimum, maximum and operational fluorescence yields commonly used in fluorescence studies: F_O_, F_M_, F_S_, F_M_′ (Kolber et al. 1998). These curve fit parameters then support calculation of absolute, rather than relative, ETR (Suggett et al. 2009; Perkins et al. 2010) and the proportion of PSII reaction centres that are instantaneously closed, referred to as the excitation pressure (1–q_P_) (van Kooten and Snel 1990). RLC techniques typically examine the responses of ETR to sequentially increasing or decreasing light intensities (Perkins et al. 2006, 2010). In the present study, however, we used a non-sequential RLC methodology (Fig. 1) to elucidate photoacclimation processes including upregulation of ETR and downregulation through regulated and non-regulated excitation dissipation, Y(NPQ) and Y(NO), respectively (Klughammer and Schreiber 2008). We hypothesised that (1) upregulation of electron transport would be represented through an increase in ETR as a function of immediate light history, (2) σ_PSII_′ would change proportionally with incident light intensity, (3) ρ would decrease as light intensity increased and hence would not counteract a decrease in σ_PSII_′ (Xu et al. 2017), and (4) in order to maximise efficiency of photoprotection, Y(NPQ) would be induced in preference to Y(NO).

## Materials and methods

Two strains of *H. ostrearia* were isolated from the west coast of France at the Île de Ré and Baie de Bourgneuf in 2015. Cells were collected by micropipette and grown in f/2 medium (Guillard and Ryther 1962). Growth and successful selection of *H. ostrearia* were monitored by light microscopy. A single strain of *H. silbo* was isolated from North Carolina, USA, also in 2015, with cells extracted and grown in the same manner. Once sufficient single species biomass had been obtained, cultures were transferred to the laboratory at Mount Allison University, Sackville, Canada, where all measurements were performed. Cultures were grown in f/2 medium (Guillard and Ryther 1962) at 20 °C and at a light intensity of 50 µmol photons m^−2^s^−1^, with a light/dark cycle of 12 h:12 h. Growth of cultures was monitored by following fluorescence emission at 680 nm using a plate spectrofluorometer (SpectraMax Gemini EM, Molecular Devices, Sunnyvale, USA).

When cultures reached mid-exponential phase, culture subsamples were taken for chlorophyll fluorescence induction measurements, by placing them in a 2 ml cuvette and then dark-adapting them for ~ 2 min. Samples were then exposed to a series of 30 s exposures to changing light levels (Fig. 1) to result in a non-sequential rapid light curve (RLC). At the end of each 30 s exposure we applied a train of 40 blue (455 nm) flashlets with a duration of 1.2 µs each separated by an intervening interval of 1.0 µs of darkness to induce an FRRf fluorescence induction curve (Kolber et al. 1998), using a Photon Systems Instruments FL3500 fluorometer system (Brno, Czech Republic). This train of 40 blue flashlets cumulatively induced a single turnover of PSII, which reduced Q_A_ to Q_A_^−^ and thereby photochemically closed PSII. Flashlet intensity was selected to saturate the fluorescence rise within around 30 of 40 flashlets (Laney 2003; Laney and Letelier 2008).

For each FRRf induction curve, data were exported from the FluorWin data capture software to fit a model with four parameters: minimal fluorescence, F_0_; maximal fluorescence, F_M_; effective absorption cross section for PSII photochemistry, σ_PSII_; coefficient of excitonic connectivity ρ; and the rapid and slow kinetic phase lifetimes for PSII reopening, τ_1 and_ τ_2_, respectively (Kolber et al. 1998) using the PSIWORX-R package (A. Barnett, sourceforge.net) (Murphy et al. 2016; Ni et al. 2016). For each measurement we applied an FRRf induction before and then again after a 1 s period of darkness to allow PSII to reopen after illumination. We thus determined in the dark F_0_, F_M_, σ_PSII_, ρ; under actinic light F_S_, F_M_ʹ, σ_PSII_ʹ ρʹ; and following 1 s of darkness after actinic light F_0_ ʹ1s, F_M_ ʹ1s, ρʹ1s, σ_PSII_ʹ1s. Note that the prime superscript indicates that the parameter was measured under actinic light whereas ′1s indicates a measurement taken from cells in instantaneous darkness immediately following actinic light.

We then estimated F_0_ʹ as:

${{\text{F}}_0}^{\prime }{\text{ }}={\text{ }}{{\text{F}}_0}{^{\prime }_{{\text{1s}}}}\; \times \;\left\{ {{\text{1}} - \left[ {{{\left( {{{\text{F}}_{\text{M}}}{{^{\prime }}_{{\text{1s}}}} - {{\text{F}}_{\text{M}}}^{\prime }} \right)} \mathord{\left/ {\vphantom {{\left( {{{\text{F}}_{\text{M}}}{{^{\prime }}_{{\text{1s}}}} - {{\text{F}}_{\text{M}}}^{\prime }} \right)} {{{\text{F}}_{\text{M}}}{{^{\prime }}_{{\text{1s}}}}}}} \right. \kern-0pt} {{{\text{F}}_{\text{M}}}{{^{\prime }}_{{\text{1s}}}}}}} \right]} \right\}$ (Oxborough and Baker 1997).

We estimated the coefficient of photochemical quenching (q_P_):

${{\text{q}}_{\text{P}}}={\text{ }}{{\left( {{{\text{F}}_{\text{M}}}^{\prime } - {{\text{F}}_{\text{S}}}} \right)} \mathord{\left/ {\vphantom {{\left( {{{\text{F}}_{\text{M}}}^{\prime } - {{\text{F}}_{\text{S}}}} \right)} {\left( {{{\text{F}}_{\text{M}}}^{\prime } - {{\text{F}}_0}^{\prime }} \right)}}} \right. \kern-0pt} {\left( {{{\text{F}}_{\text{M}}}^{\prime } - {{\text{F}}_0}^{\prime }} \right)}}$ (Kramer et al. 2004).

We estimated the yield of non-photochemical quenching (Y[NPQ]):

${\text{Y}}\left( {{\text{NPQ}}} \right)={\text{ }}{{\text{F}}_{\text{S}}}/{\text{ }}{{\text{F}}_{\text{M}}}^{\prime }{\text{ }} - {\text{ }}{{\text{F}}_{\text{S}}}/{{\text{F}}_{\text{M}}}$ (Klughammer and Schreiber 2008),

and the yield of non-regulated excitation dissipation (Y[NO]):

Y(NO) = F_s_/F_M_ (Klughammer and Schreiber 2008).

For plotting the non-sequential light curves, we calculated the ETR as:

$${\text{ETR}}={{{\text{E }}\; \times \;{\sigma _{{\text{PSII}}}}^{\prime }{\text{ }} \times {\text{ Y}}\left( {{\text{PSII}}} \right)} \mathord{\left/ {\vphantom {{{\text{E }}\; \times \;{\sigma _{{\text{PSII}}}}^{\prime }{\text{ }} \times {\text{ Y}}\left( {{\text{PSII}}} \right)} {\left( {\left( {{{\text{F}}_{\text{M}}} - {\text{ }}{{\text{F}}_0}} \right)/{{\text{F}}_{\text{M}}}} \right)}}} \right. \kern-0pt} {\left( {\left( {{{\text{F}}_{\text{M}}} - {\text{ }}{{\text{F}}_0}} \right)/{{\text{F}}_{\text{M}}}} \right)}},$$

where Y(PSII) is the quantum efficiency of PSII calculated as (F_M_ʹ–F_S_)/F_M_ʹ.

This is modified from Suggett et al. (2009) as we did not include n_PSII_ (usually set to 0.002) and we expressed ETR as e− PSII^−1^ s^−1^. In some cases our light response curve extended to actinic light levels high enough to suppress the remaining variable fluorescence to small values when FRRf inductions were performed in the presence of actinic light. Statistical analyses and plotting were performed using R Version 3.3.2 (R Core Team 2016) with the package ‘minpack.lm’ (Timur et al. 2016).

In order to determine the interaction of regulated and non-regulated excitation dissipation, regulated excitation dissipation Y(NPQ) was inhibited in replicates treated with DL-dithiothreitol (DTT, Bioshop), which inhibits xanthophyll cycling through suppression of the de-epoxidation of diadinoxanthin to diatoxanthin (Bilger and Björkman 1990; Lavaud et al. 2002; Ni et al. 2016). DTT was prepared as fresh 5 mM stock on each day of measurements dissolved in ethanol. 20 µL aliquots of the stock were added to 2 ml of culture to produce a final concentration of 5 µM which was reported to fully inhibit Stern–Volmer non-photochemical quenching (NPQ) (Ni et al. 2016). These treatments were then examined using the same non-sequential RLC as described above, with five replicate RLCs for each *Haslea* strain.

Finally, the same strains of *Haslea* were exposed to induction–recovery curves whereby subsamples from each culture, as described above were exposed to actinic light (induction phase) prior to a dark recovery period. Two light levels were used, 300 and 600 µmol photons m^−2^ s^−1^ to represent comparatively lower and higher light stress and hence different levels of hypothesised upregulation of ETR and induction of excitation dissipation. The induction phase lasted 1200 s (20 min) and was followed by a dark recovery phase of 900 s (15 min), to investigate the time course of induction and reversal of upregulation and excitation dissipation, respectively. Measurements were made using the FRRf fluorometer and data analysis procedures as described for the RLCs described above.

## Results

**Fig. 2:**
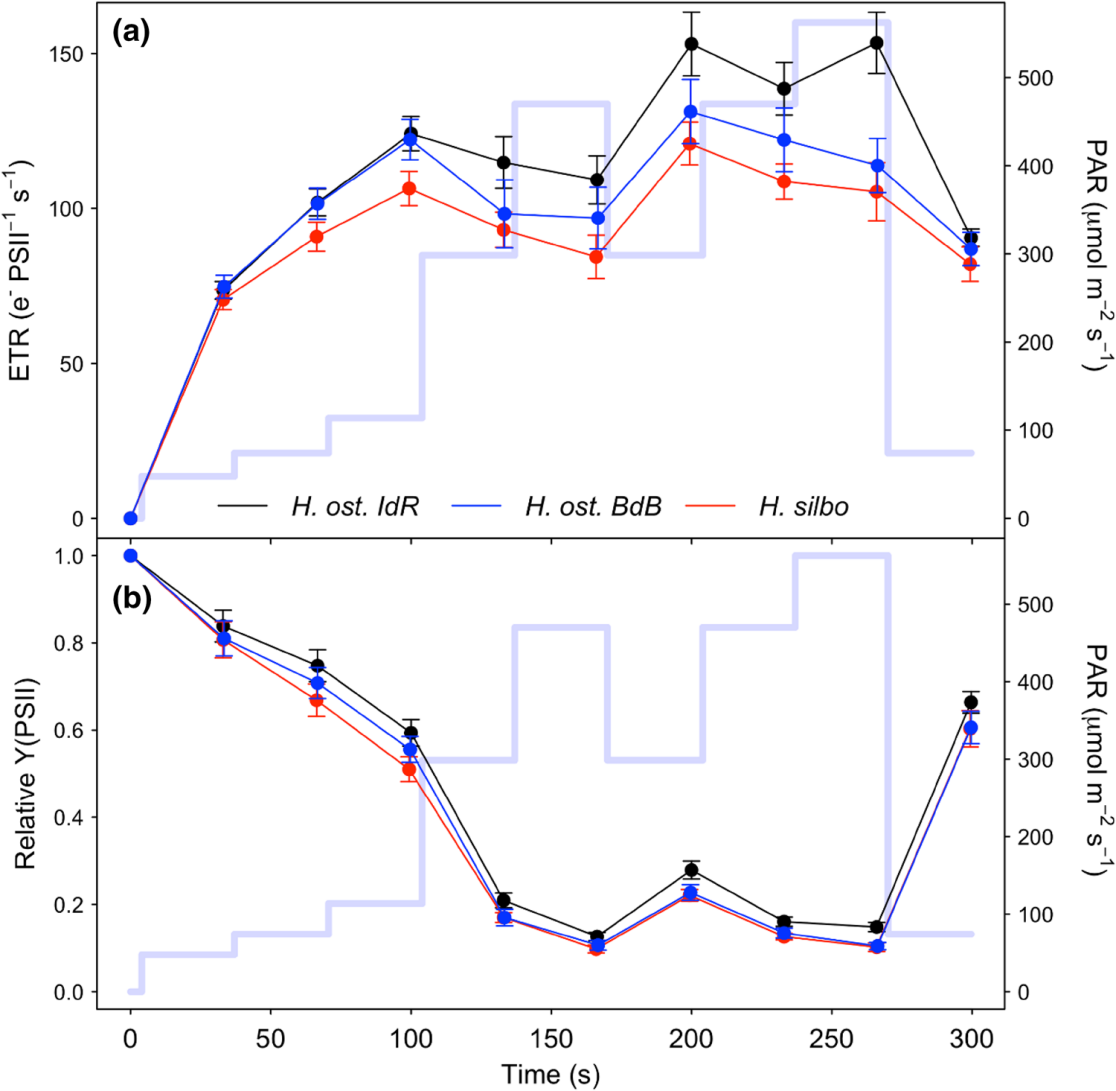
Photosystem II (**a**) ETR and (**b**) quantum yield (Y) over the course of non-sequential rapid light curves for *H. ostrearia* strain Île de Ré (*H. ost. IdR*), *H. ostrearia* strain Baie de Bourgneuf (*H. ost. BdB*), and *H. silbo* (mean ± SE, *n* = 3). Background trace (light blue line) shows the respective light levels (right axes) applied at each light step

**Fig. 3:**
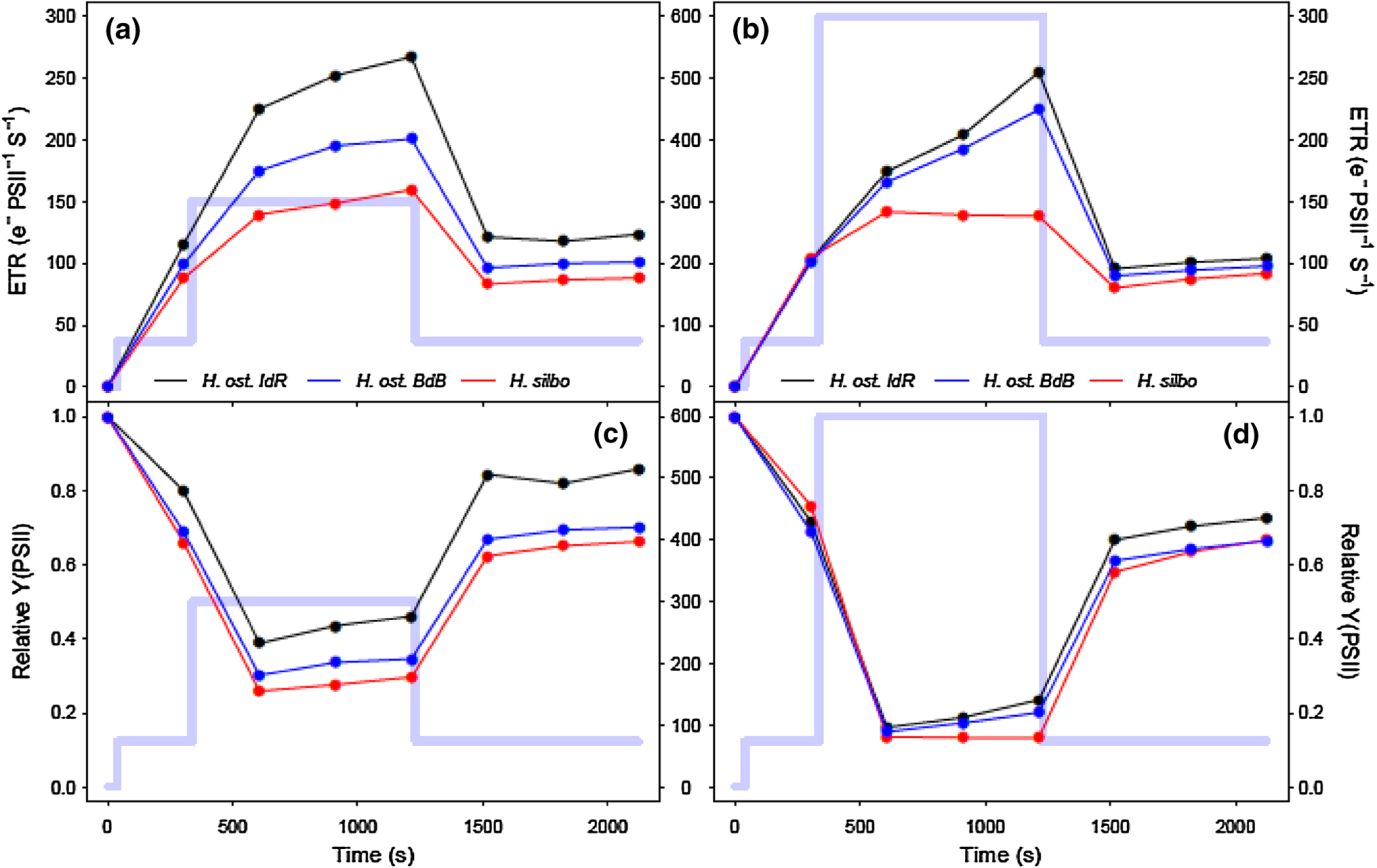
Photosystem II (**a, b**) ETR and (**c, d**) quantum efficiency (Y) over the course of induction and recovery curves at low light (left column) and high light (right column) for *H. ostrearia* strain Île de Ré (*H. ost. IdR*), *H. ostrearia* strain Baie de Bourgneuf (*H. ost. BdB*), and *H. silbo* sp. nov. ined.. Background trace (light blue line) shows the respective light levels (central axes, PAR µmol photons m^−2^ s^−1^) applied at each light step

All three *Haslea* strains showed similar patterns of rapid photoacclimation in ETR (Fig. 2a) and photosystem II quantum efficiency (Y(PSII); Fig. 2b) during non-sequential rapid light curves (RLCs). ETR increased for the first three increasing light steps up to PAR 114 µmol m^−2^ s^−1^, before a downward curvature showing supersaturation for the fourth and fifth light curve steps up to a PAR of 470 µmol m^−2^ s^−1^, 180 s into the light curve. Subsequently, during the remainder of the non-sequential light curve steps, ETR and Y(PSII) departed from proportional changes with PAR. When PAR was lowered from 470 back to 300 µmol m^−2^ s^−1^, ETR and Y(PSII) increased to values greater than previously measured at this light level during the initial incrementally increasing PAR phase of the light curve. Subsequently, when PAR was reincreased back to 470 and on to 540 µmol m^−2^ s^−1^, ETR and Y(PSII) increased to values greater than those measured previously, and hence greater than the values of ETR at which the light curves had previously saturated (Fig. 2a). Thus, ETR at a given high light level increased in response to the light history during the short 30 s light curve steps of the RLC. After 270 s, PAR was lowered to the same level as the second light step of the RLC (i.e. ~ growth irradiance, 74 µmol m^−2^ s^−1^), whereby ETR decreased to a value slightly lower than the original value initially measured at this light level, and Y(PSII) went to a value that was slightly lower than the original value. During the induction phase of the induction–recovery curves (Fig. 3), ETR increased for the lower light treatment (Fig. 3a) and for two of the three strains in the high light treatment (Fig. 3b), with the exception of *H. silbo*, attributable to an increase in Y(PSII) over time at elevated light (Fig. 3c,d).

**Fig. 4:**
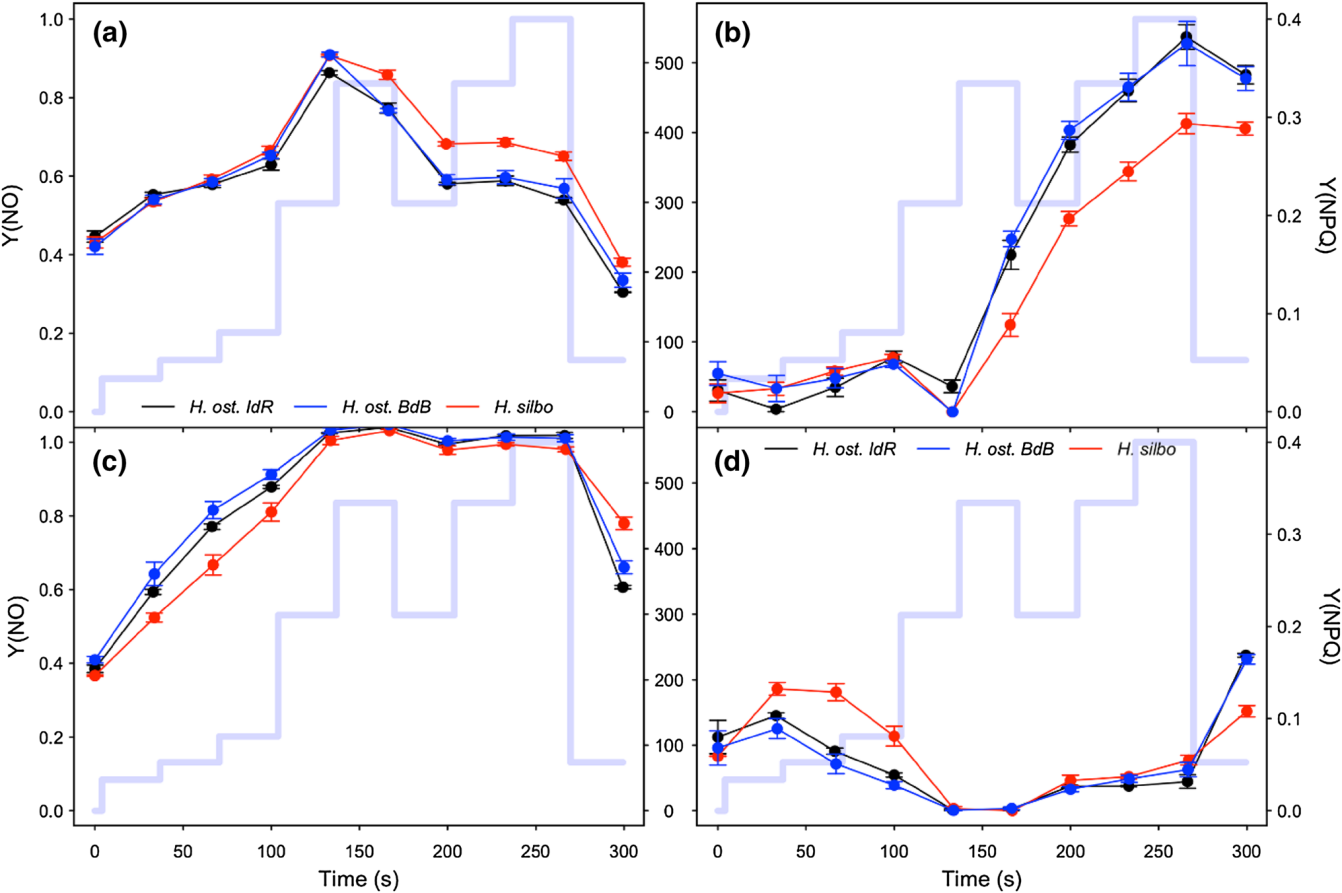
Excitation dissipation responses of *H. ostrearia* strain Île de Ré (*H. ost. IdR*), *H. ostrearia* strain Baie de Bourgneuf (*H. ost. BdB*), and *H. silbo* sp. nov. ined., during non-sequential rapid light curves (mean ± SE, *n* = 3). Non-regulated (Y[NO], **a** and **c**) and regulated (Y[NPQ], b and d) excitation dissipation components are shown, for untreated samples (**a** and **b**), and samples treated with DTT (**c** and **d**). Background trace (light blue line) shows the respective light levels (middle axes, PAR µmol photons m^−2^ s^−1^) applied at each light step

**Fig. 5:**
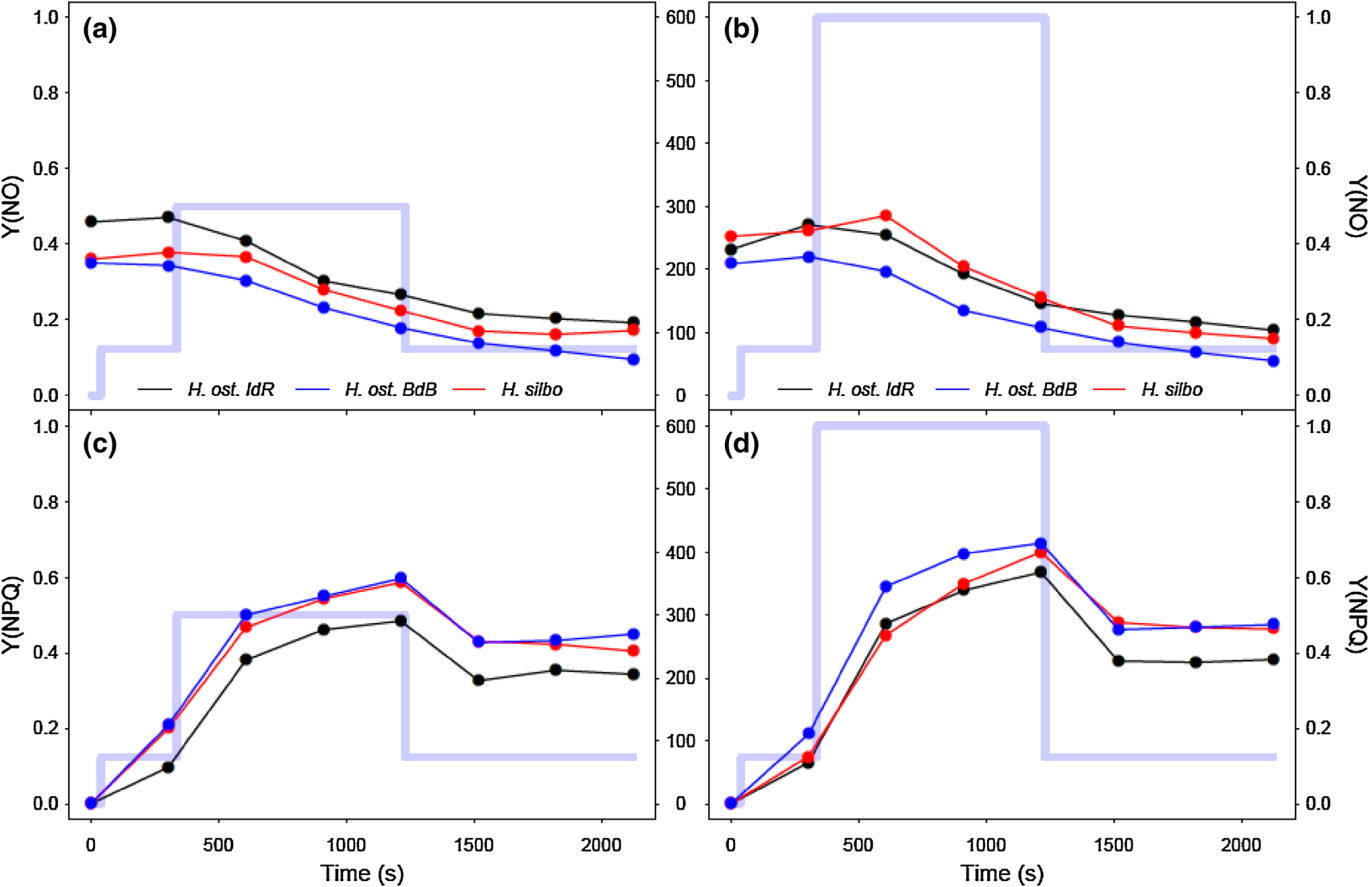
Excitation dissipation responses of *H. ostrearia* strain Île de Ré (*H. ost. IdR*), *H. ostrearia* strain Baie de Bourgneuf (*H. ost. BdB*), and *H. silbo* sp. nov. ined., during low light (left column) and high light (right column) induction and recovery curves. Both non-regulated (Y[NO], **a, b**) and regulated (Y[NPQ], **c, d**) excitation dissipation components are shown. Background trace (light blue line) shows the respective light levels (central axes, PAR µmol photons m^−2^ s^−1^) applied at each light step

All three strains of *Haslea* showed similar excitation dissipation responses to changing light, with non-regulated excitation dissipation Y(NO) (Fig. 4a) increasing from ~ 0.4 in darkness to ~ 0.85 as PAR increased for the first four sequential RLC steps up to 300 µmol photons m^−2^ s^−1^. Coincident with the onset of saturation of ETR (~ 300 µmol photons m^−2^ s^−1^; Fig. 2a), regulated, energy-dependent, excitation dissipation Y(NPQ) was induced (Fig. 4b), with a concomitant decline in Y(NO) (Fig. 4a). Once induced, Y(NPQ) increased progressively with *time* under high light, as opposed to reflecting patterns in PAR during the non-sequential phase of the RLC. At the end of the RLC, Y(NPQ) remained elevated whilst Y(NO) declined to less than initial values, despite the return to growth irradiance. This time-dependent induction of Y(NPQ) was confirmed with induction–recovery curves (Fig. 5), whereby decreases in Y(NO) in both lower and higher light induction phases (Fig. 5 a, b) paralleled steady induction of Y(NPQ) (Fig. 5c, d). Note that in both light treatments, incomplete reversal of Y(NPQ) during the dark recovery phase was apparent. In notable contrast, RLC treatments with DTT addition showed a steady increase in Y(NO) to saturation at ~ 1 during the first four incremental increases in PAR (Fig. 4c), whilst Y(NPQ) decreased (Fig. 4d); the latter a function of increasing F_M_′ (data not shown) above initial values in the dark, suggesting residual NPQ at the start of the RLC. DTT treatment thus served to suppress Y(NPQ) induction, and consequently Y(NO) was not inhibited.

**Fig. 6:**
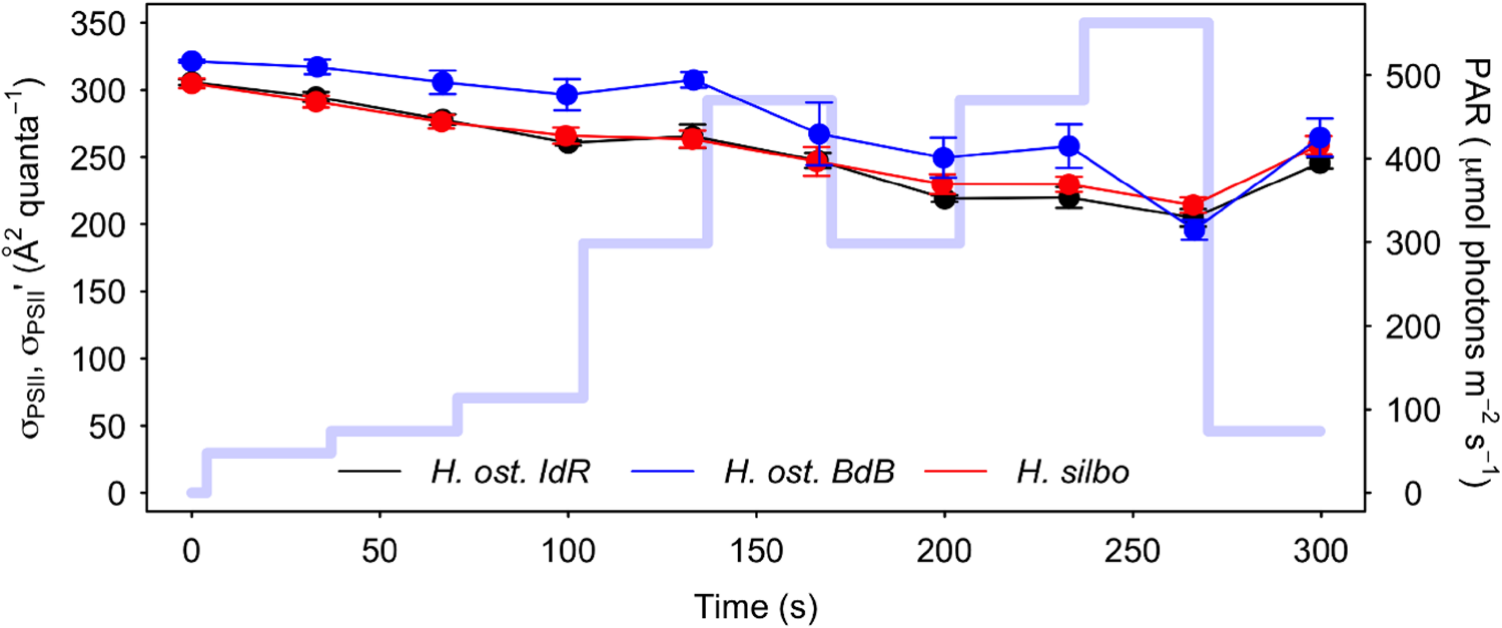
Photosystem II (PSII) effective absorption cross section (σ_PSII_, σ_PSII_′) of *H. ostrearia* strain Île de Ré (*H. ost. IdR*), *H. ostrearia* strain Baie de Bourgneuf (*H. ost. BdB*), and *H. silbo*, during non-sequential rapid light curves (mean ± SE, *n* = 3). Background trace (light blue line) shows the respective light levels (right axes) applied at each light step

The cross-sectional absorbance of PSII, σ_PSII_′, decreased throughout the course of RLCs (Fig. 6.), in parallel with a decrease in reaction centre connectivity, ρ (Supplementary Fig. S1), as light level and reaction centre closure (1–qP) increased. Across all RLC data, σ_PSII_′ showed a negative relationship to Y(NPQ) (Supplementary Fig. S2a), whereas no clear relationship was apparent between σ_PSII_′ and Y(NO) (Supplementary Fig. S2b), nor to the sum of NPQ parameters (Supplementary Fig. S2c). Across all induction/recovery curve data, normalised σ_PSII_′ decreased as a linear function of increasing Y(NPQ) (*R*^2^ = 0.43, *P* < 0.001, *n* = 48, Fig. Supplementary Fig. S3); however, this response was less than unity (slope = − 0.65), indicating that increases in Y(NPQ) did not drive proportional decreases in σ_PSII_′. Overall, with Y(NPQ) induction over RLCs, the time-dependent upregulation of ETR was not driven by corresponding increases in σ_PSII_′ nor ρ′, and hence another process was responsible for the increases in Y(PSII) and ETR.

**Fig. 7:**
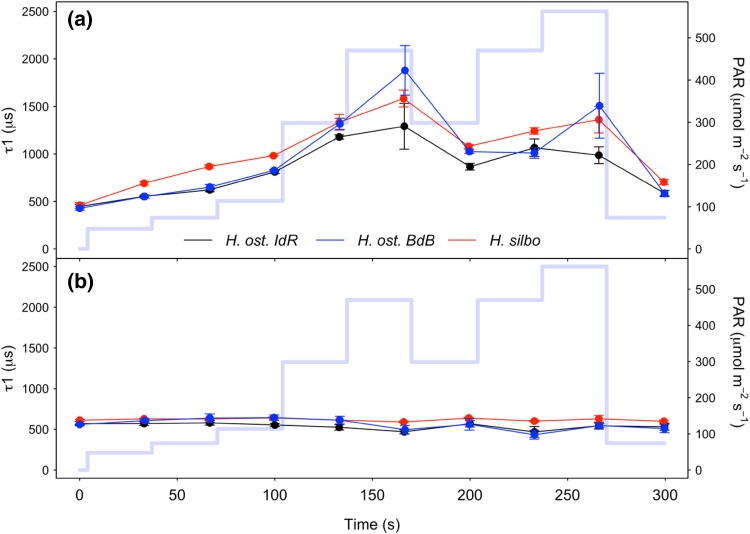
τ_1_ reaction centre opening (Q_A_− oxidation) lifetimes for *H. ostrearia* strain Île de Ré (*H. ost. IdR*), *H. ostrearia* strain Baie de Bourgneuf (*H. ost. BdB*), and *H. silbo*, measured during non-sequential rapid light curves (**a**) in the presence of actinic light at the end of each 30 s light step, and (**b**) following 1 s darkness immediately after exposure to each preceding light step (mean ± SE, *n* = 3). Background trace (light blue line) shows the respective light levels (right axes) applied at each light step

**Fig. 8:**
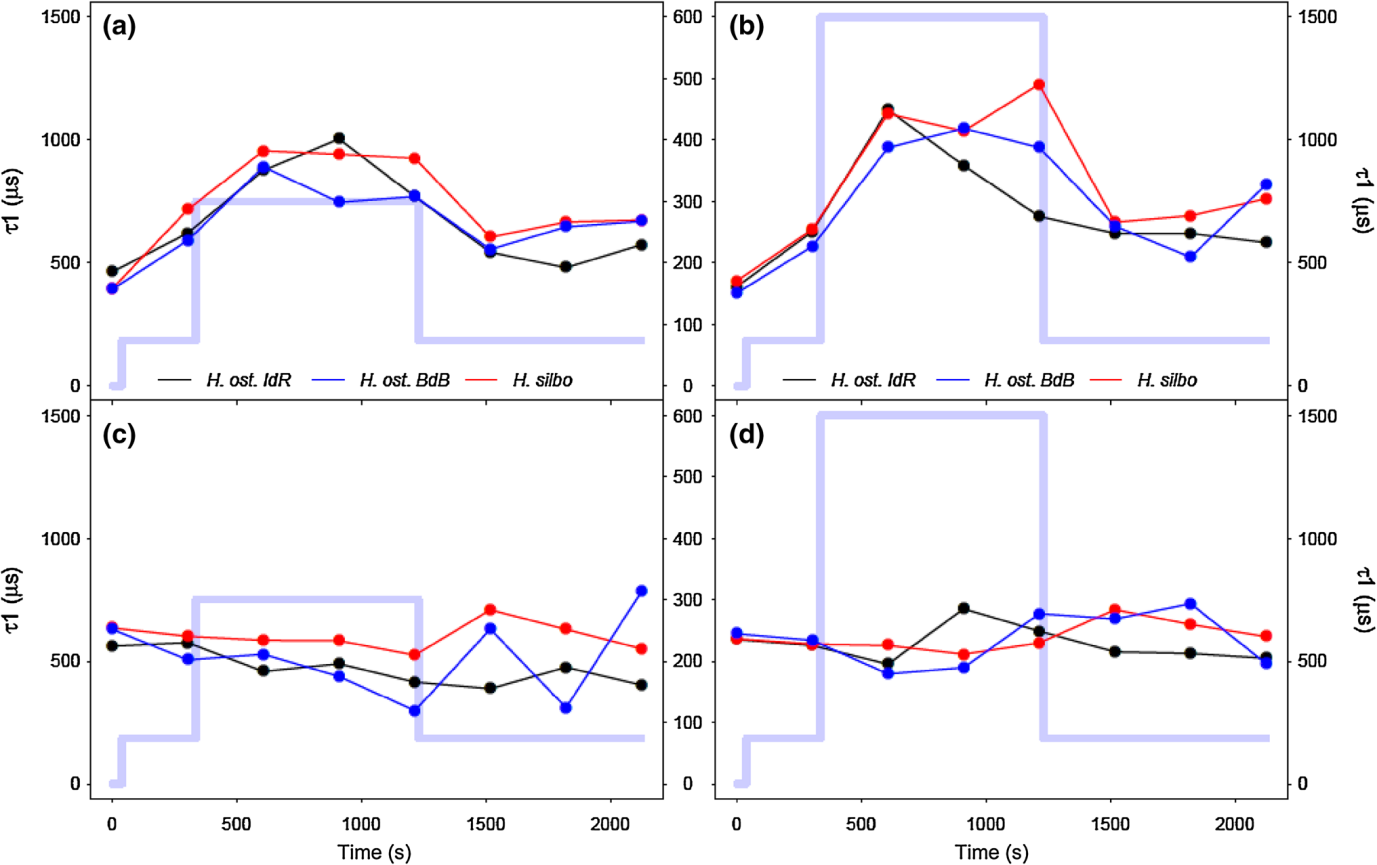
τ_1_ reaction centre opening (Q_A_− oxidation) lifetimes for *H. ostrearia* strain Île de Ré (*H. ost. IdR*), *H. ostrearia* strain Baie de Bourgneuf (*H. ost. BdB*), and *H. silbo*, measured during low light (left column) and high light (right column) induction and recovery curves (**a, b**) in the presence of actinic light at the end of each 30 s light step, and (**c, d**) following 1 s darkness immediately after exposure to each preceding light step. Background trace (light blue line) shows the respective light levels (central axes, PAR µmol photons m^−2^ s^−1^) applied at each light step

With exposure to increasing PAR during initial RLC steps up to 470 µmol photons m^−2^ s^−1^, reaction centre opening lifetimes (τ_1_) measured in the light increased from ~ 500 to ~ 1800 µs as excitation pressure increasingly exceeded downstream electron transport (Fig. 7a). Upon subsequent decrease in PAR to 300 µmol photons m^−2^ s^−1^, τ_1_ decreased to just 1000 µs, below previous values measured at the same PAR. Thereafter, τ_1_ remained accelerated despite subsequent reexposure to high PAR, indicating that the initial exposure to high light had induced process(es) that accelerated electron transport away from PSII. This mechanism was retained whilst samples remained under high light exposure, but was lost when samples were returned to growth irradiance during the final RLC step. Notably, τ_1_ lifetimes measured after only 1 s of darkness (Fig. 7b) remained stable at ~ 500 µs throughout the duration of non-sequential light response treatments and thus dynamism in τ_1_ appeared strictly dependent upon exposure to high illumination. Under induction/recovery curves (Fig. 8), again τ_1_ generally decreased in a time-dependent manner following exposure to induction phase irradiances, with *H. silbo* remaining an exception under high light, while stable τ_1_ lifetimes were apparent at ~ 500 µs when measured after 1 s darkness. Overall, examination of the relationship between Y(PSII) and τ_1_ across all datasets revealed a significant negative relationship (*R*^2^ = 0.76, *P* < 0.0001, *n* = 78, Supplementary Fig. S4), indicating that shorter lifetimes for Q_A_ oxidation, and hence more rapid oxidation on the acceptor side of photosystem II (PSII), drove higher quantum efficiencies of PSII, which in turn supported the light history-dependent acceleration of ETR (Fig. 2).

## Discussion

*Haslea* diatoms showed rapid photoacclimation dependent upon their immediate light history. ETR that initially saturated during incrementally increasing light steps of RLCs was subsequently upregulated through stepped changes in light intensity. Upregulation of relative ETR (rETR, in the absence of measurement of light absorption) has previously been reported in diatoms, but was assumed to be a light proportional response (e.g. Perkins et al. 2006). Indeed, an upregulation of ETR may have contributed to failure of light curve saturation during a number of previous studies, including those performed in situ with diatom-dominated estuarine biofilms (Perkins et al. 2002), cyanobacterial cryoconite communities (Perkins et al. 2017), or during *ex situ* assessment of Streptophyte microalgae (Yallop et al. 2012), although the role of cell motility and forms of physiological downregulation through use of tertiary pigments are also known contributing factors in these studies. Similarly time-dependent induction of excitation dissipation and/or non-photochemical quenching has also been reported in situ for microalgae (Perkins et al. 2017). Increases in maximum ETR with extended illumination during RLCs has previously been reported in vascular plants (White and Critchley 1999) and chlorophyte macroalgae (Ihnken et al. 2010). However, the present study is the first to investigate mechanisms driving such upregulation, and to highlight the time-dependent nature of this process, concomitant with the induction of regulated excitation dissipation.

The high light upregulation of ETR identified in this study was not explicable in terms of a change in σ_PSII_′, which actually decreased in response to progressive induction of regulated excitation dissipation, Y(NPQ), over the same time periods. Additionally, reaction centre excitonic connectivity (ρ, which actually decreased) did not serve to offset the decreases in σ_PSII_′. Instead, the increases in ETR were explicable by a decrease in the τ_1_ lifetime for reopening of PSII. This acceleration of downstream electron transport was dependent upon exposure to high light, and relaxed within 1 s of darkness, or under lower light. We suspect that this acceleration reflects the opening of additional electron flux(es) when the plastoquinone (PQ) becomes reduced under high excitation pressure. The patterns of upregulated ETR and Y(PSII), in response to preceding exposure to high light can therefore be explained by the increased rate of electron transport away from PSII, detected by analyses of PSII reopening after a single turnover saturating flashlet (Fig. 1) (McCauley and Melis 1987; Cao and Govindjee 1990; Kolber et al. 1998). This reopening can be fitted with 2 or 3 kinetic phases, of which the quickest (τ_1_), with a lifetime on the order of ~ 500 µs (Cao and Govindjee 1990), reflects electron flow from Q_A_^−^ to Q_B_ in PSII centres that already carry a bound plastoquinone (Q_B_) at the time of the flashlet. Such centres can pass electrons from Q_A_^−^, reduced during the single turnover flashlet, to the bound Q_B_ in a rapid electron transfer, giving a rapid photochemical reopening (Fig. 1). Our data show a decrease in the lifetime for the rapid oxidation of Q_A_^−^ through electron flow to Q_B_ (τ_1_) dependent upon short-term light history. Importantly, this increase in rate of electron transport, once induced, was retained even when the applied actinic light level was decreased, so long as light remained above the initial level of light saturation. We hypothesise either upregulation of ETR through an increase in linear electron transport and in turn increased productivity, or alternatively a photoprotective upregulation through the use of alternative electron acceptor(s), e.g. oxygen-dependent electron transport. Due, however, to the rapid reversibility of the acceleration in τ_1_, we suspect photoprotective flow(s) to alternative acceptors, in particular oxygen-dependent electron transport, rather than increased production and utilisation of reducing agents further downstream. Either process, however, demonstrates a competitive advantage in diatoms to rapidly upregulate photochemistry either as a form of photoprotection when exposed to high light, or as a real increase in photosynthetic productivity to exploit high light (Curien et al. 2016; Gerotto et al. 2016; Ilík et al. 2017).

Downregulation of PSII was monitored through both non-regulated Y(NO) and regulated Y(NPQ), energy dissipation. To minimise damaging impacts of high light exposure, a low ratio of Y(NO) to Y(NPQ) may be beneficial (Klughammer and Schreiber 2008). In this study, we observed initial induction of Y(NO) at low light intensities, but once a threshold in light intensity, or possibly light dose, was achieved which corresponded to initial saturation of ETR, regulated Y(NPQ) was induced and Y(NO) decreased. Similar patterns have been observed in other eukaryotic microalgae, but not in photosynthetic prokaryotes (Xu et al. 2017). The threshold for Y(NPQ) induction has been suggested to correspond to an excitation pressure (1-q_P_) of approximately 45% (Ruban et al. 2004; Lavaud et al. 2007), but this does not appear to be a constant for all diatoms (Lavaud et al. 2016). It would seem likely that previous culture light history will at least in part determine the threshold for the induction of regulated downregulation and the rate of induction above a threshold (Lavaud et al. 2016), as reported by Xu et al. (2017). In this study, above ~ 305 µmol m^−2^ s^−1^ PAR, Y(NPQ) induction became time dependent rather than light intensity dependent. Lavaud and Goss (2014) provide a model for non-photochemical quenching in diatoms, proposing a two-stage process of xanthophyll conversion, de-epoxidation of diadinoxanthin to diatoxanthin, followed by disassociation of fucoxanthin–chlorophyll protein complexes (FCPs) from PSII to act as isolated energy quenchers. This two-stage NPQ model indicates different time scales for induction and relaxation of NPQ that would be unable to respond quickly to the changes in PAR, hence once NPQ was induced, it would not relax with short decreases in light intensity. This would explain the persistence of Y(NPQ) despite reduction in light observed in this study. Such a process seems sensible for cells exposed to repeated rapid changes in light intensity, e.g. within the pelagic and benthic zones of an estuary, where light intensity may rapidly increase and decrease. Lavaud and Goss (2014) also report the ability of diatoms to retain active diatoxanthin in the dark, in readiness for exposure to light and hence a retained capacity for instant downregulation (e.g. Perkins et al. 2002; Serôdio et al. 2005b).

Regulated excitation dissipation in the form of Y(NPQ) was probably the dominant cause of a decrease in PSII cross section, σ_PSII_′, observed for *Haslea* diatoms in this study. σ_PSII_′ measures the outcome of two processes, the capture of light by the light-harvesting complex associated with PSII, and the subsequent quantum yield of photochemistry for open PSII reaction centres (Kolber et al. 1998; Suggett et al. 2009). In a study of two prokaryote and two eukaryote taxa of microbial phototrophs, Xu et al. (2017) reported no relationship between σ_PSII_′ and Y(NPQ) for the prokaryotes at light levels above the light saturation coefficient, Es. However for the eukaryotes, they reported a progressive decline in σ_PSII_′ as Y(NPQ) increased, although the decrease in normalised σ_PSII_′ was proportionally smaller than the increase in Y(NPQ). Xu et al. (2017) further investigated the possibility that this relationship was affected by reaction centre connectivity, i.e. does an increase in $\rho$ counteract the decrease in σ_PSII_′ induced by Y(NPQ), but reported no influence on σ_PSII_′ by ρ. This agrees with our findings whereby the increase in Y(NPQ) produced a significant decrease in σ_PSII_′ and a concomitant decrease in ρ, as excitation pressure (1–qP) increased. Our data demonstrated the presence of an initial decrease in σ_PSII_′ due to reaction centre closure, followed by a greater rate of decrease in σ_PSII_′ once Y(NPQ) is induced above a light threshold and, presumably, a threshold of excitation pressure. This has important impacts on the calculation of ETR utilising σ_PSII_′ without taking into account changes in the latter as light intensity increases. Goss and Lepetit (2015) reported the high capacity of diatoms for NPQ induction and we would therefore expect diatoms, per se, to exhibit a large decrease in σ_PSII_′ as light intensity increases, as observed in the present study.

In summary, *Haslea* diatoms are capable of rapid photoacclimation that alters the relation between ETR and light intensity (PAR), such that ETR is upregulated depending on immediate light history. Furthermore, this increase in ETR occurs despite a concomitant drop in σ_PSII_′ caused by induction of energy-dependent excitation dissipation Y(NPQ). Declines in σ_PSII_′ are not compensated for by changes in reaction centre connectivity (ρ), as the latter also decreases with increasing light intensity. The induction of regulated excitation dissipation (Y[NPQ]) after a threshold of light exposure is reached, results in inhibition of non-regulated quenching (Y[NO]). These findings have important consequences for our interpretation of photophysiological parameters, and demonstrate the selective advantage of these diatoms in fluctuating light environments through induction of regulated forms of both photochemical and non-photochemical energy dissipation.

## Electronic supplementary material

Below is the link to the electronic supplementary material.

Supplementary material 1 (DOCX 521 KB)
