# Supplementary material for: Time-dependent upregulation of electron transport with concomitant induction of regulated excitation dissipation in Haslea diatoms
Source: Photosynth Res. 2018 Apr 16;137(3):377–88. doi: 10.1007/s11120-018-0508-x (PMC6182385; doi:10.1007/s11120-018-0508-x)
Supplement: Supplementary file 1 — Supplementary material 1 (DOCX 521 KB) [file 11120_2018_508_MOESM1_ESM.docx]

**Perkins et al. Supplementary Figures (Figures S1 to S4)**


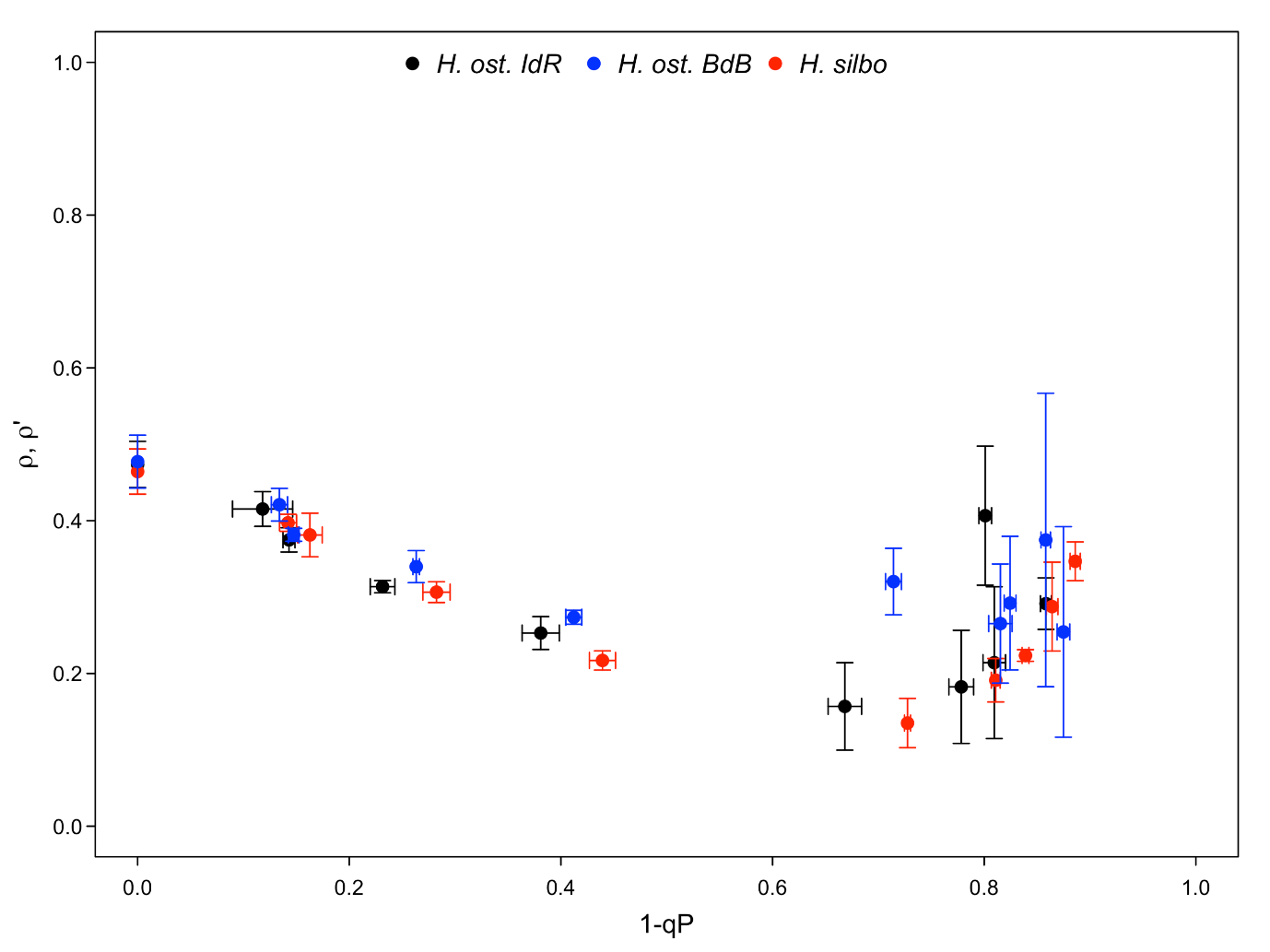


Figure S1. Reaction centre connectivity (ρ, ρ’) as a function of excitation pressure (1-qP) for *Haslea ostrearia* strain Île de Ré (*H. ost. IdR*), *H. ostrearia* strain Baie de Bourgneuf (*H. ost. BdB*), and *H. silbo* (mean ± SE, n = 3).


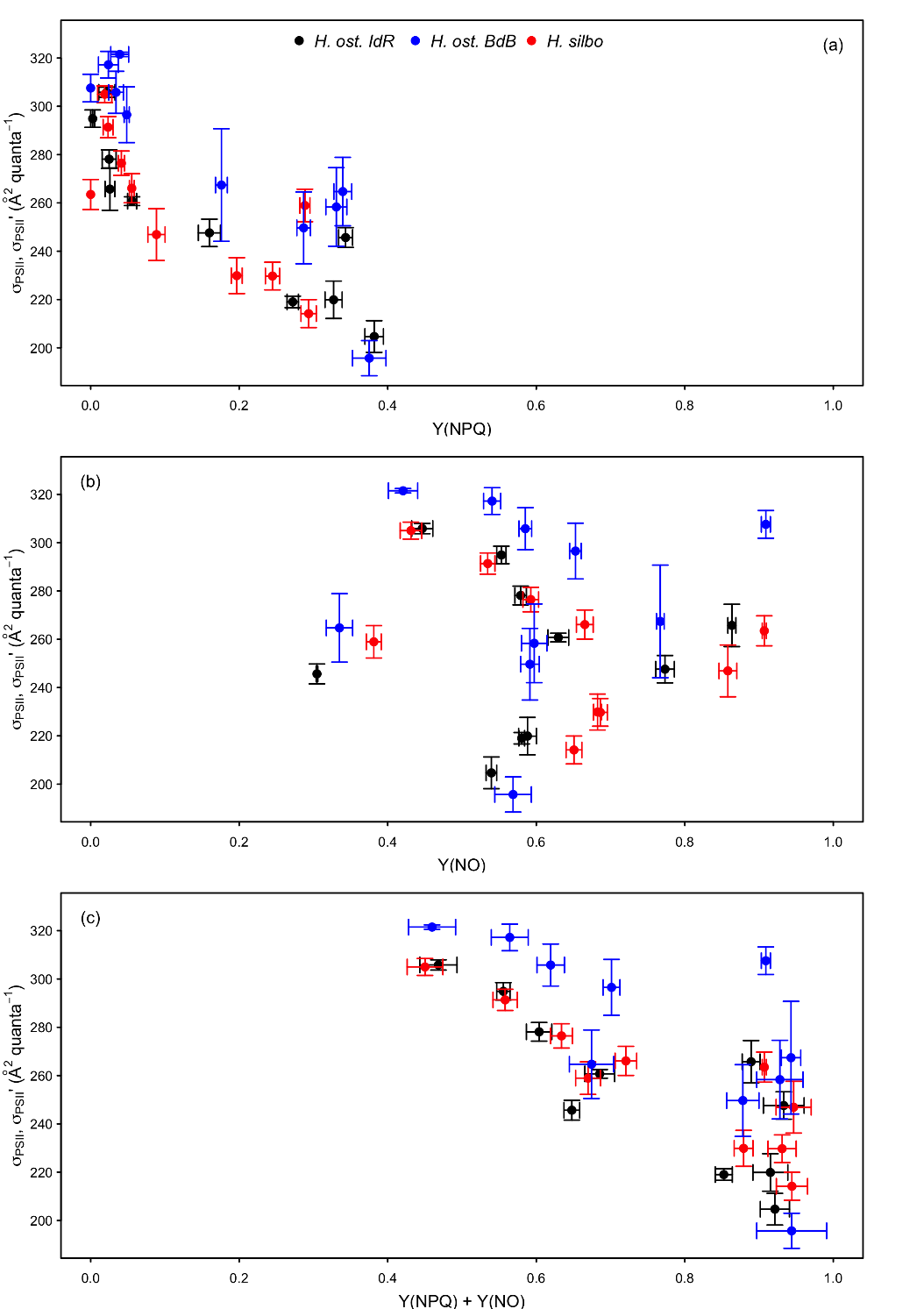


Figure S2. Photosystem II (PSII) effective absorption cross section (σ_PSII_, σ_PSII_’) as a function of (a) regulated excitation dissipation (Y[NPQ]), (b) non-regulated excitation dissipation (Y[NO]), and (c) the sum of regulated and non-regulated excitation dissipation, for *Haslea ostrearia* strain Île de Ré (*H. ost. IdR*), *H. ostrearia* strain Baie de Bourgneuf (*H. ost. BdB*), and *H. silbo* (non-sequential light curve data, mean ± SE, n = 3).

Figure S3: Normalized σPSII or σPSII’ vs. Y(NPQ) derived from induction/recovery data, showing regression line (black line), 95 % confidence intervals (dash red lines), and the associated coefficient of determination (R^2^). The black dotted line demonstrates unity between the two variables.

Figure S4: Y(PSII) vs. τ_1_ derived across all rapid light curve (red points) and induction/recovery curve (black points) data, showing the regression line (black line), 95 % confidence intervals (red dashed lines) and the associated coefficient of determination (R^2^).
